# Management of diversity and inbreeding when importing new stock into an inbred population

**DOI:** 10.1093/jhered/esad027

**Published:** 2023-04-29

**Authors:** Brian Kinghorn, Alexander Kinghorn

**Affiliations:** School of Environmental and Rural Science, University of New England, Armidale, NSW, Australia; School of Environmental and Rural Science, University of New England, Armidale, NSW, Australia

**Keywords:** genetic diversity, mate selection, optimal contributions selection

## Abstract

This article relates to breeding programs that seek to manage genetic diversity. The method maximizes a multicomponent objective function, applicable across breeding scenarios. However, this paper focuses on breeding decisions following immigration of 10 unrelated individuals into a highly inbred simulated population (*F* ≈ 0.34). We use Optimal Contribution Selection to maximize retention of genetic diversity. However, some treatments add Coancestry Assortative Mating (*CAM*). This helps to avoid early dilution of immigrant genetic material, maximizing its ability to contribute to genetic diversity in the longer term. After 20 generations, this resulted in considerably increased genetic diversity, with mean coancestries 59% of what random pairing gave. To manage progeny inbreeding, common practice is to reject matings above an upper limit. As a suboptimal rules-based approach, this resulted in 26% *decreased* genetic diversity and 8% *increased* inbreeding in the long term, compared with random pairing. In contrast, including mean progeny inbreeding as a continuous variable in the overall objective function decreased final inbreeding by 37% compared with random pairing. Adding some emphasis on selection for a single trait resulted in a similar pattern of effects on coancestry and inbreeding, with 12% higher trait response under *CAM*. Results indicate the properties of alternative methods, but we encourage users to do their own investigations of particular scenarios, such as including inbreeding depression. Practical implementation of these methods is discussed: they have been widely adopted in domestic animal breeding and are highly flexible to accommodate a wide range of technical and logistical objectives and constraints.

## Introduction

When an ex situ captive population has become highly inbred and suffers low genetic diversity, a small number of “unrelated” and possibly “outbred” individuals from the wild or a captive population can be introduced to help rectify this. There could be more diversity in the small introduced group than in the whole of the inbred population, although this is not required. Random pairing in this newly combined population means that the new genetic material can be quickly diluted by the existing genetic material, with loss of opportunity for the new genetic material to make suitably high contributions in the longer term (Lacy 1994; Ballou et al. 2010).

However, the rules-based Ranked *MK* Selection method (Ivy and Lacy 2012), as commonly implemented in *PMx* software (Traylor-Holzer 2011), preferentially pairs immigrant animals to other immigrant animals, because they are of low mean kinship (*MK*) to the full population. This solves the immigration problem by helping to extend the period of introgression of the new genetic material into the overall population.

The imported genetic material will have been built up to higher animal numbers without losing its high concentration through frequent crossing into the home population. Overall, this delayed and/or reduced crossing means that higher levels of dissemination of the new genetic material can be reached without resorting to ongoing importations. This is illustrated in Supplementary Materials through use of pedigree diagrams. Eventually, the newly imported genetic material will become well intermingled with the original genetic material. However, using this approach, the imported genetic material will have had greater opportunity to reach the ideal levels of contribution for each immigrant, to maximize genetic diversity. The impact of doing this has not yet been illustrated, but will be in this paper.

In practical implementation of a zoo/conservation breeding program, many component objectives need to be accommodated, in addition to management of genetic diversity. These include management of progeny inbreeding, health and behavior issues, transport and other costs, plus genetic issues such as managing Mendelian conditions, and selection on 1 or more traits. Use of a rules-based approach to selection and mating decisions becomes problematic with such component objectives. For example, the setting of threshold conditions, whereby a mating is rejected because it breaks a declared limit, is suboptimal: matings that are well away from the limit do not benefit from that high margin, whereas matings that just escape rejection are equally accepted. Both must compete equally for the rules on the next issue. The effects of this will be shown strongly in this paper.

Such sequential decision-making can lead to suboptimal outcomes, even within the single component of managing genetic diversity. This is illustrated by example in Supplementary Material for Ranked *MK* Selection.

To solve this issue, we use a generalized method for implementing breeding programs that is not rules-based, but integrates all technical and logistical issues in a single objective function to be optimized (Kinghorn and Shepherd 1999; Kinghorn et al. 2008; Kinghorn 2011; Kinghorn and Kinghorn 2023). These issues compete with each other under control by the practitioner, to arrive at mating recommendations for practical implementation. In this project, we need a generalized method to integrate different components:

- Selections that give low population mean coancestry.
- Matings that extend availability of immigrant genetic material.
- Matings that reduce progeny inbreeding.
- Inclusion of some emphasis on a competing priority—trait selection.

The objectives of this paper are:

1. To use the generalized method to solve the immigration problem (by implementing Coancestry Assortative Mating [*CAM*] within that method).
2. To illustrate the impact of using this method to manage long-term genetic diversity in the immigration scenario.
3. To illustrate the impact of adding a competing priority to the breeding goal—selection for a single trait.
4. To compare Progeny Inbreeding management under the generalized method against the commonly used rules-based approach for avoiding Progeny Inbreeding.

## Methods

### Mean kinship and mean coancestry

Zoo breeding practitioners and software commonly use the term “kinship” whereas domestic animal and plant breeders and software commonly use “coancestry.” These terms are equivalent and used interchangeably in this paper—they are both a measure of relationship between 2 individuals, and equal the probability that 2 alleles, 1 randomly chosen from each individual, are identical by descent.

*MK*_*i*_ is classically defined as the average of the kinship coefficients between individual *i* and all living individuals (including itself) in the population (Ballou and Lacy 1995; Ballou et al. 2010). However, when planning breeding operations to improve genetic diversity, our *MK*_*i*_ calculations should ideally target the average of the kinship coefficients between an individual and all members of the *future* population (including its descendants). For this we need to exclude living animals that will not be used for breeding, and we need to account for increased contributions from any animals that are to be widely used. This means that in practice, a slightly different version of mean kinship is needed, excluding nonbreeding individuals, which we term *MK*_*i*_, and this is recalculated during analyses that generate new animal pairing recommendations. This is already being done for individual animals at each iteration in commonly used *MK* Selection methods (Ivy and Lacy 2012). It also means that *MK*_*i*_ values used in such analyses depend on analysis method.

*MK*_*i*_ should be calculated for all animals in a manner that best reflects impact of the final list of pairings on future genetic diversity. Accordingly, in its simplest form under discrete generations, *MK*_*i*_ in this paper is the mean kinship or coancestry of individual *i* across all members of the *selected* population, including itself, weighted by the number of mating allocations for *i*. This weighting is 1 for single-pair mating, but more where the solution dictates that a male is to be used as the mate for multiple females. *MK*_*i*_ is also weighted according to number of mating allocations made to all other animals, as the number of allocations to *i*’s relatives affects *MK*_*i*_. Under overlapping generations, we also account for potential future contributions from juveniles and other currently unavailable animals, as described later. The average *MK*_*i*_ across the population is often referred to as Mean Parental Coancestry, calculated as *cʹAc*/2, where *c* is the vector of animal contributions for the current mating cycle, summing to 1, and *A* is the matrix of numerator relationships (2 × coancestries) among all candidates (Meuwissen 1997).

### Optimal contributions selection

The methods used in this paper follow Optimal Contribution Selection (*OCS*, Meuwissen 1997). Where management of genetic diversity is the only objective, and where no relationship information is available, then, as expected, *OCS* aims to minimize Mean Parental Coancestry, and this drives the genetic contribution of each candidate animal within each sex toward equality. With the addition of relationship information, the contributions change accordingly. For example, if there are just 3 males, 2 full sibs and 1 unrelated male, the unrelated male is allocated more females than each of the 2 full sibs, with a ratio of 3:2:2 (Kinghorn et al. 2008; see also https://youtu.be/2ARCANK6rdA?t=180). The unrelated male’s coancestry with the full selected population is increased due to his own higher contribution. In fact, the mean coancestry for each selected candidate is driven to equality, maximizing retention of genetic diversity.

Where the objective is a balance of genetic diversity and some merit factor(s), such as increased genetic merit or reduced capture and transport costs, then the higher contribution allocated to a high-merit candidate, which reduces genetic diversity, is compensated for under *OCS* by altering contributions from other candidates, to “re-balance” genetic diversity. This means, for example, that a target level of population mean coancestry can be maintained while maximizing other aspects of merit.

In our implementation, optimal contributions are not calculated, as in Meuwissen (1997), but arrived at by consequence of maximizing objective functions that aim to increase genetic diversity and/or genetic gains for chosen traits or multitrait indices. In addition to this, the objective function adds parameters that dictate mate allocations, such as reducing progeny inbreeding and inhibiting the mating of carriers of recessive genetic conditions. This is not a 2-step procedure, but a combined portfolio approach that optimizes all immediately required selection and mating decisions simultaneously, using an evolutionary algorithm (Kinghorn and Shepherd 1999; Kinghorn 2011; Kinghorn and Kinghorn 2023).

### Coancestry Assortative Mating

**Fig. 1.**
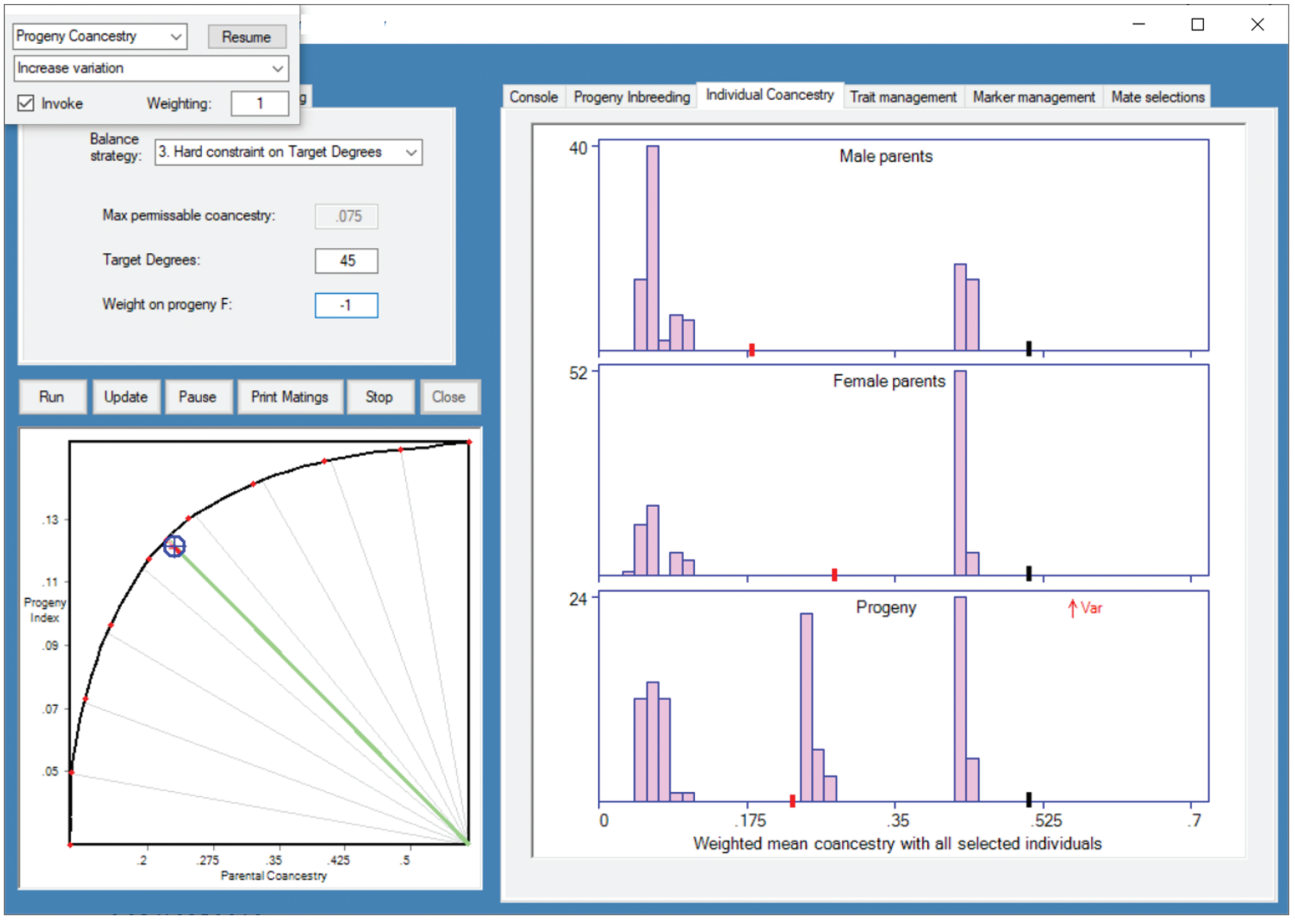
Program for optimizing mate selection (Kinghorn and Kinghorn 2023). Treatment is *OCS*_*td*_*CAM.F*_1.0_ with an initial population at $\bar{F}\approx 0.95$, and the year is mating in 2023, for progeny in 2024. The lower-left graph shows the frontier of possible outcomes for genetic gain (Progeny Index = mean index of predicted progeny EBVs across traits) and genetic diversity (Mean Parental Coancestry). The current objective targets 45 degrees on this frontier—equal emphasis on gain and diversity. The blue target icon shows the value of the current solution (mating list). The lower-right histogram shows distribution of mean coancestries or kinships (*MK*_*i*_) for the prospective progeny. This distribution is multimodal, as expected due to importing outside breeding stock in 2021, and the objective function includes a weighting of +1 on increased variation (small window at top-left).

This paper introduces *CAM*, invoked by adding the parameter “variation in the progeny distribution of mean coancestry/kinship” as a component of the overall objective function to be optimized. Appropriate weighting on this component of the objective function promotes solutions that show assortative mating on coancestry/*MK*_*i*_, with high-to-high *MK*_*i*_ matings and low-to-low *MK*_*i*_ matings among selected candidates, increasing variation in progeny coancestry values, as seen in Fig. 1. This results in interbreeding among immigrants (low-*MK*_*i*_), and among their descendants in later generations, with increasing crossing into the main population as the number of their descendants increases through selection advantages. Maintaining this higher concentration of immigrant genetic material in the population extends its ability to inject diversity into the main population over generations, as illustrated in Supplementary Fig. S6, and as seen in the results.

*CAM* is implemented with calculation of an *MK*_*i*_ value for each individual candidate, *i*, against just the *selected* candidates in the current solution, including weightings according to the number of matings allocated to each candidate. In addition, the CAM component of the objective function is not over-riding. It must compete with all other issues in the objective function. However, in this paper the only competing issue that affects mate allocation is Progeny Inbreeding management, such that interpretations can be made with some clarity.

### Management of progeny inbreeding

The common practice of rejecting matings that exceed a threshold inbreeding coefficient affects mate allocations, and this can disrupt *CAM* and degrade these favorable outcomes. However, the alternative policy of weighting against mean progeny inbreeding in the overall objective function competes with *CAM* at the level of individual matings, meaning that key matings of high value to *CAM* can persist in the face of relatively high progeny inbreeding values. This paper will test whether an appropriate weighting on mean progeny inbreeding can give good outcomes for both genetic diversity and progeny inbreeding.

### Population data generated in simulations

The population simulation program PopSim (Cowling et al. 2017) was used to generate and propagate breeding populations. This program is highly configurable, but parameters were set to simple values to help give clear illustration in this paper: No repeat matings in either sex, each male can mate a maximum of 2 females, exactly 4 progeny produced by each selected breeding female, no mortality, a single trait simulated under the infinitesimal model (Barton et al. 2017), with *h*^2^ = 0.25 and fixed and covariable effects set to zero. Coancestry and inbreeding parameters were calculated from the known pedigree, which was also used to calculate BLUP estimates of breeding value (*EBV*) for the trait. Please see Cowling et al. (2017) for details on methods. A generation is taken to be 1 yr, for simple illustration. Results presented for all treatments are the mean of 5 replicate runs. The population structure and settings adopted are not supposed to represent any particular species: we generated a somewhat idealized population in order to make reasonably clear contrast of the treatments invoked. In particular, results in this paper should not be taken as recommendations, but as indicators of the properties of the methods tested. This program is available for those wishing to test species-typical parameters instead. At each year, PopSim was directed to use the method and code of Kinghorn and Kinghorn (2023) for selection and mating decisions, as described below.

Preparation phase, years 2000 to 2021 (exactly the same for all treatments): This is for generating an initial population at $\bar{F}\approx 0.34$. The approach to create initial populations at $\bar{F}\approx 0.49$ and $\bar{F}\approx 0.95$ is similar:

1. Starting in year 2000 from a population of unrelated individuals, 32 single-mate pairs were used each year, giving population $\bar{MK}=0.3376$ and mean inbreeding $\bar{F}=0.3394$ in year 2018.
2. From 2018, all female progeny were bred, to double population size each year, but selecting as of year 2020 to cap populations size at 100 breeding females per year thereafter, resulting in $\bar{MK}=0.3384$ and $\bar{F}=0.3352$ in year 2021. This is a moderately big population that is in danger because of its low diversity and high inbreeding level.
3. In year 2022, 5 unrelated males plus 5 unrelated females were introduced as candidates for selection. These were individuals of unknown prior pedigree, assumed to be noninbred and unrelated to all individuals in the main population. This was simply achieved by setting the parents of 5 male and 5 female progeny candidates to “unknown” in the simulation, leaving trait values as typical of the main population. The test is from this prepared point, before selection in year 2022.

Treatment phase, years 2022 to 2040: Selection and mating each year using the treatments described below.

### Experimental treatments

The treatments were different methods of constructing a mating list to be used at each year in the simulations. Treatments were as follows, where *x* = *d* for a target of high *d*iversity alone and *x* = *td* for a combined target of favorable genetic change for the *t*rait simulated and high *d*iversity:

**Table:** 

*OCS*_*x*_ *R*	Optimal Contribution Selection, Random pairing
*OCS*_*x*_ *R.F*_*y*_	Optimal Contribution Selection, Random pairing except for a weighting value *y* to reduce progeny inbreeding
*OCS*_*x*_ *CAM*	Optimal Contribution Selection, Coancestry Assortative Mating
*OCS*_*x*_ *CAM.F*_*y*_	Optimal Contribution Selection, Coancestry Assortative Mating with weighting value *y* to reduce progeny inbreeding
*OCS*_*x*_ *CAM.F*_*Limit*_	Optimal Contribution Selection, Coancestry Assortative Mating with rejection of matings with inbreeding above *F*_*limit*_

For *OCS*_*x*_*CAM.F*_*Limit*_ we applied an “*F* upper limit” as specified under *Auto Pair* in *PMx* software (Traylor-Holzer 2011, p. 89), to reject any mating above *F*_*limit*_. We followed the default *PMx* limit policy with limits adjusted upwards as the population mean F increases, but in a continuous fashion, with *F*_*limit*_ set just below the minimum *F* value across all possible half-sib (or more closely related) matings among the prevailing candidates. Notice that this “sliding scale” is only used to choose *F*_*limit*_, which is then applied as a fixed parameter across all prospective matings in the prevailing year of analysis.

The combined target of favorable genetic change for the trait simulated and high diversity (*td*) was implemented by using an intermediate 45 degrees on the frontier of possible outcomes for each selection round, where 0 degrees represents full emphasis on high genetic gain using *EBVs* for the simulated trait, and 90 degrees represents full emphasis on high genetic diversity, or low $\bar{MK}$ (Fig. 1, also see, e.g., Cowling et al. 2017). The method used to approach optimal selection and mating solutions for these treatments is based on Kinghorn (2011), which employs an adaptation of Differential Evolution (Storn and Price 1997) to maximize the prevailing objective function. A note on scaling of input parameters is provided in Supplementary Material. The actual implementation and its availability for research are described in detail by Kinghorn and Kinghorn (2023).

## Results

**Fig. 2.**
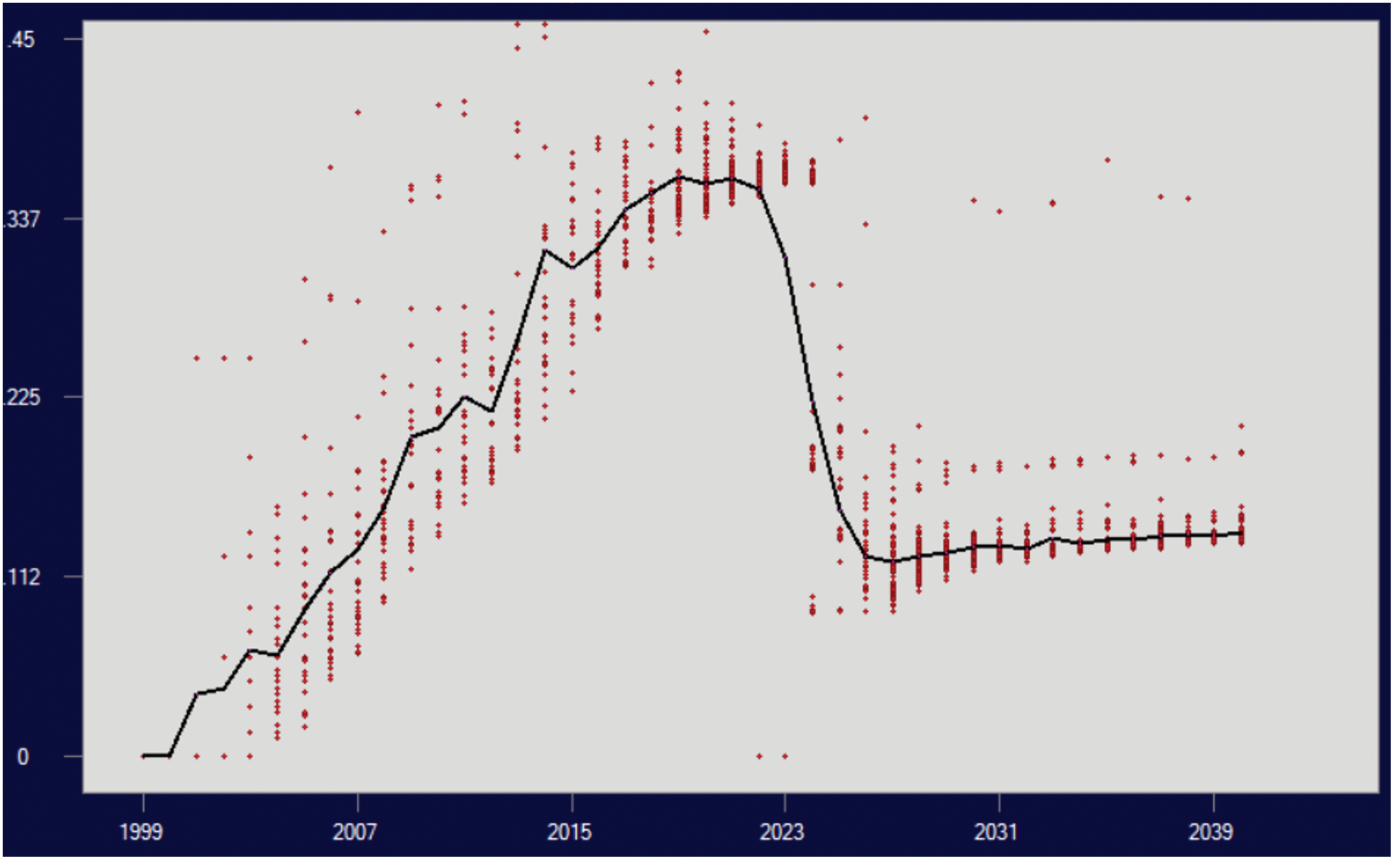
PopSim graph for a single replicate of treatment *OCS*_*d*_*R*. Progeny inbreeding is plotted against birth year (red dots for individuals, and black line for year means). In years 2006 to 2018 use of just 32 mating pairs per year results in high inbreeding levels. Over years 2018 to 2020 population size is increased to 100 breeding females. In year 2022, 10 unrelated individuals were introduced, and the population was bred using *OCS*_*d*_*R*. Some zero inbreeding levels can be seen following importation, as expected. Full- and half-sib matings can be seen, especially in in later years, as red dots in 2 groups well above the black line, as *OCS*_*d*_*R* does not avoid these.

Figure 2 shows results from 1 replicate PopSim run of treatment *OCS*_*td*_*R*. This shows the buildup of inbreeding during the preparation phase, a marked decrease for a few years after importing outside stock, then settling down to a slow increase in inbreeding. The pattern for mean coancestry is similar, as expected. The manner in which this develops is illustrated using pedigree diagrams, in Supplemental Material (see Supplementary Fig. S6).

### Results targeting genetic diversity alone

**Fig. 3.**
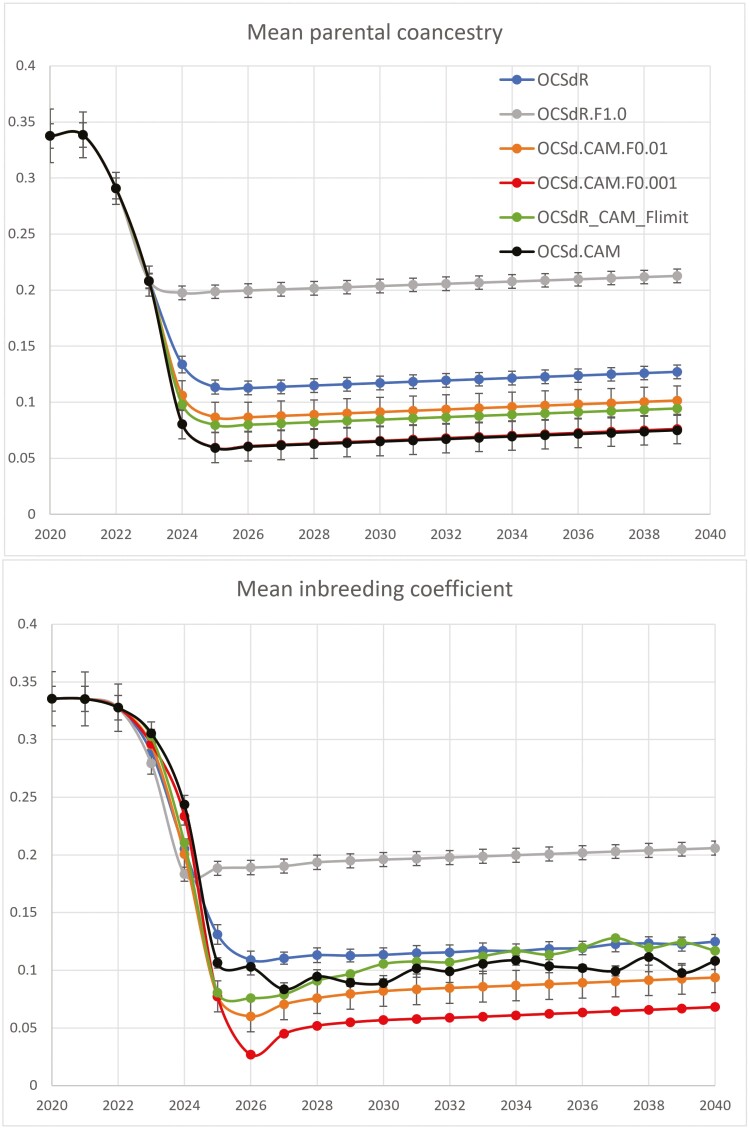
Results for treatments targeting genetic diversity alone and initial $\bar{F}=0.34$. Results for all treatments are the same up to 2020, and are shown in Fig. 2. Black overplots red in the top graph.

Figure 3 shows mean coancestry and inbreeding results for treatments targeting diversity alone (Target Degrees = 90), under the importation scenario used. *OCS*_*d*_*R* ended in year 2040 with $\bar{MK}=0.1271$ and $\bar{F}=0.1248$, but *OCS*_*d*_*CAM* was considerably better at $\bar{MK}=0.075$ and $\bar{F}=0.1081$. *OCS*_*d*_*R.F*_1.0_ initially lowered progeny inbreeding (following the *GCmc* treatment of Fernández and Caballero 2001), but ended with much higher values of $\bar{MK}=0.2127$ and $\bar{F}=0.2059$. This was promoted by mating between immigrants and the main population to lower progeny inbreeding, and thus losing opportunity to optimally exploit new genetic material over generations.

*OCS*_*d*_ *R.F*_1.0_ and *OCS*_*d*_*R.F*_0.1_ (not shown) gave the same results, indicating very good convergence in the mate selection algorithm: These 2 treatments targeted the same optimal outcome (at which inbreeding avoidance did not impact on animal selection), but by following 2 different routes, resulting in the same individuals selected and the same allocations made to give the same results, over 5 replicates × 34 generations = 170 separate analyses per treatment. Average runtime to convergence for an analysis (400 candidates) was 4.8 min.

*OCS*_*d*_ *CAM.F*_0.01_ initially lowered progeny inbreeding compared with *OCS*_*d*_*CAM*, but followed *OCS*_*d*_*R.F*_1.0_ to some extent, as the 0.01 weighting on *F* detracted sufficiently from emphasis on *CAM*, and worked against it. Lowering this weighting in *OCS*_*d*_*CAM.F*_0.001_ allowed *CAM* to impact almost fully on coancestry ($\bar{MK}=0.0761$ vs. 0.0750 for *OCS*_*d*_*CAM*), but with sufficient residual impact on mate allocations to lower progeny inbreeding values considerably ($\bar{F}=0.0681$ vs. 0.1081). The value in this small emphasis to reduce inbreeding is seen in years 2022 to 2025, when population structure is such that there are groups of candidate matings of equivalent value for *CAM*, but of different value for progeny inbreeding. This small benefit is not seen in mean coancestry results, as, unlike progeny inbreeding, mate allocation does not affect mean coancestry in the following generation. The favorable long-term results for *OCS*_*d*_*CAM.F*_0.001_ over *OCS*_*d*_*CAM.F*_0.01_ could be tempered somewhat in the face of inbreeding depression, which was not included in these simulations, as inbreeding level under *OCS*_*d*_*CAM.F*_0.001_ was higher in 2024 (0.2336 vs. 0.2006, Fig. 3). This would likely affect fertility.

Treatment *OCS*_*d*_*CAM.F*_*Limit*_, which rejects matings above *F*_*Limit*_, was less effective at lowering coancestry than *OCS*_*d*_*CAM.F*_0.001_ or *OCS*_*d*_*CAM*, probably because of elimination of key half-sib matings that the CAM approach promotes. This treatment performs even more poorly for mean progeny inbreeding, and, as for *OCS*_*d*_*CAM*, $\bar{F}$ fluctuates notably over generations. This is probably because patterns in the relationship within pairs allocated according to their *MK*_*i*_ values, is only partially moderated by the *F*_*limit*_ threshold, which only operates at the high end of the progeny *F* distribution. As can be seen, the policies that place a weighting on $\bar{F}$ control these fluctuations rather well.

### Results targeting both diversity and trait change

**Fig. 4.**
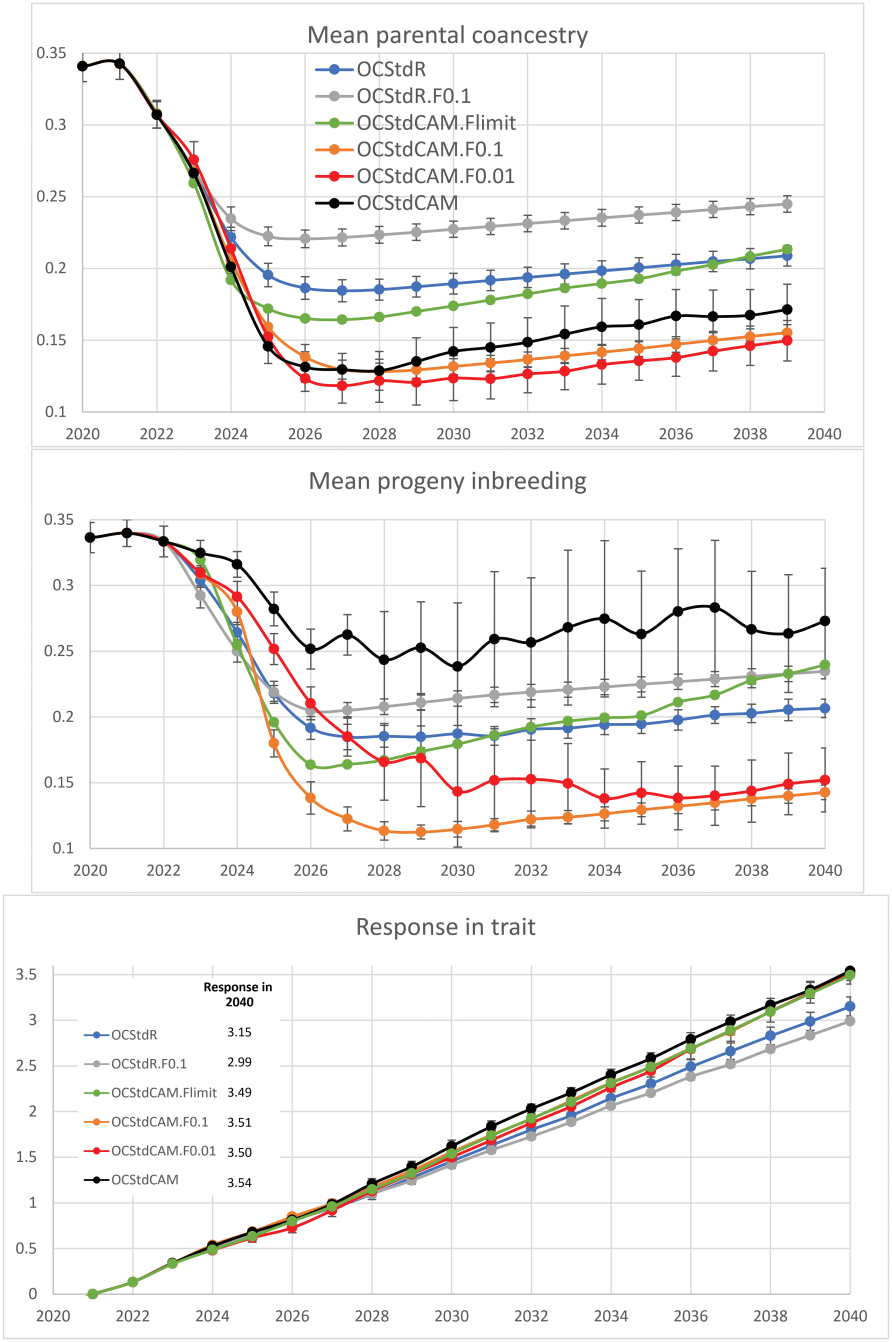
Results over years for treatments that place equal emphasis on genetic diversity and genetic gain and initial $\bar{F}=0.34$. Error bars are ±1 standard error. Green largely overplots orange in the bottom graph.

Figure 4 shows coancestry, inbreeding and genetic gain (selection response in the trait) for treatments targeting both genetic gain and genetic diversity, under the importation scenario adopted. *OCS*_*td*_*R* ended with $\bar{MK}=0.2088$, but *OCS*_*td*_*CAM* was notably better at $\bar{MK}=0.1714$. *OCS*_*td*_*CAM.F*_0.01_ and *OCS*_*td*_*CAM.F*_0.001_ improved on this with $\bar{MK}=0.1553$ and 0.1497, as described below.

With a fixed threshold policy, *OCS*_*td*_*CAM.F*_*limit*_ will reject some *CAM*-favored matings in early generations, unabated by attention to trait selection. Although this is bad for longer-term diversity, it is probably favorable for short-term diversity, with wider use of imported individuals across the whole population. This might help explain low coancestry in 2023 and 2024 under *OCS*_*td*_*CAM.F*_*limit*_, but higher coancestry than the other *CAM* treatments thereafter.

A notable result is the high mean and variation between replicates for $\bar{F}$ under *OCS*_*td*_*CAM*. This is most probably driven by the fact that high-*MK*_*i*_ individuals tend to be related to each other, as do low-*MK*_*i*_ individuals. For pedigree *MK*_*i*_, full sibs have identical *MK*_*i*_ values. Thus, with no constraint on progeny inbreeding, assortative mating on *MK*_*i*_ results in high progeny inbreeding, despite low parental coancestry. This is more prevalent when there is trait selection, because coancestry values are more free to increase in mean and variation, giving more opportunity to allocate mates that are highly related.

To manage this high inbreeding, stronger weights on $\bar{F}$ are required compared with no trait selection. *OCS*_*td*_*CAM.F*_0.1_ manages progeny inbreeding strongly (Fig. 4). It also improves coancestry compared with *OCS*_*td*_*CAM*, reducing the incidence of high-*MK*_*i*_ to high-*MK*_*i*_ matings between close relatives in favor of high-*MK*_*i*_ to high-*MK*_*i*_ matings between less related individuals, and thus improving diversity. But the weighting of 0.1 on $\bar{F}$ seems to overdo this—the gentler touch of *OCS*_*td*_*CAM.F*_0.01_ beats *OCS*_*td*_*CAM.F*_0.1_ with lower coancestry as from 2025, but at the cost of higher progeny inbreeding levels for many generations. As discussed later, changing weightings over time may be warranted.

The key results for managing diversity with equal emphasis on trait change are that using *CAM* in addition to *OCS* gave 82% of the coancestry of *OCS* alone, and adding a weighting of 0.01 on $\bar{F}$ in *OCS*_*td*_*CAM.F*_0.01_ decreased this figure to 72%. *OCS*_*td*_*CAM.F*_0.01_ gave just 61% of the coancestry under *OCS*_*td*_*R.F*_0.01_, due to the damaging effect of aiming to reduce progeny inbreeding in the absence of *CAM*, for the scenario simulated.

Selection response 2021 to 2040 was 3.15 phenotypic standard deviations for *OCS* alone. With extra genetic variation retained, the *CAM* treatments gave increased selection responses ranging 3.49 (*OCS*_*td*_*CAM.F*_*limit*_), through 3.51 (*OCS*_*td*_*CAM.F*_0.1_) to 3.54 (*OCS*_*td*_*CAM*) (Fig. 4). Rates of change are related to Mean Parental Coancestry, due to its effect on genetic variance, but inbreeding levels per se were not important, as expected with inbreeding depression not included in construction of trait values. *OCS*_*td*_*R.F*_0.1_ and *OCS*_*td*_*R.F*_1.0_ had low responses of 2.99, underlining the danger of incorrectly managing progeny inbreeding in this scenario.

Table 1 summarizes key results. The top-left figure shows that for this importation scenario *OCS*.*CAM* leads to a substantial increase in retention of genetic diversity, with only 59% of the Mean Coancestry achieved under *OCS* alone. An aberrant figure is 132% for mean inbreeding in the top row. This is seen in Fig. 4 and explained above. The bottom row in Table 1 shows that placing a limit on progeny inbreeding resulted in mean coancestry and inbreeding between 26% and 72% higher than where an appropriate weighting against mean progeny inbreeding is used.

**Table 1.** Summary results across initial population mean inbreeding coefficients.

	Mean coancestry		Mean inbreeding		Response
	Diversity alone	Diversity + trait	Diversity alone	Diversity + trait	Diversity + trait
*OCS.CAM.F*_0_ as % of *OCS.R.F*_0_	59	82	87	132	112
*OCS.CAM.F*_*best*_ as % of *OCS.R.F*_*best*_	59	72	55	69	112
*OCS.CAM.F*_*limit*_ as % of *OCS.CAM.F*_*best*_	126	142	172	168	99

*F*_*best*_ relates to the weighting on progeny inbreeding that gave the best result. For *OCS.R.F*_*best*_, *best* = 0 in all cases, for reasons described in the text.

### Results from using more highly inbred populations

Simulations were also made for populations that started from higher inbreeding levels ($\bar{F}\approx 0.49$ and 0.95), with detailed results shown in Supplementary figures and summary results in Supplementary Table S1. As might be expected, results are generally stronger for these higher initial inbreeding levels.

## Discussion

### Management of genetic diversity

*CAM* and *MK* Selection (Ballou et al. 2010) can both improve retention of genetic diversity under the scenario adopted. However, results show that using a fixed limit on progeny inbreeding would increase final population mean coancestry and inbreeding values by 26% and 72% (Table 1). Such a fixed limit is implemented by default with the *Auto Pair* option under *MK* Selection in *PMx* software, although there is option for users to over-ride this and accumulate sequential pairing decisions into the *Selected Pairs* table (Traylor-Holzer 2011, p. 87).

Compared with a conceptually large wild population, the loss of genetic diversity under the *OCS*_*d*_*CAM* method was just 59% of that under standard *OCS*_*d*_*R*. For an objective that includes genetic gain, this figure was 82%, reducing to 72% with appropriate control of progeny inbreeding. Moreover, selection response was 12% higher when using *CAM.*

The scenario adopted is favorable for this outcome, but it is probably a simple reflection of many zoo animal population scenarios. With importation of 1 sex alone, results would be less strong, due to the required crossing into the main population in the first generation. However, the *CAM* approach would still work, given opportunity to mate among these crosses, and to backcross them to pure immigrants under overlapping generations, if appropriate.

It may be valuable to test and/or adopt this method in any scenario that involves recent or proposed admixture between populations. However, it is unlikely to be of benefit in populations that are well established with a history of reasonably free migration, until new migrations are proposed.

There is some potential to apply the current methods to wild isolated populations, especially where inference about animal relationships can be made from visual or electronic observations and/or genomic information. In such cases, mating recommendations can be made using Mixed Mating Groups (Kinghorn and Kinghorn 2023), or similar, whereby 2 or more groups, each consisting of both males and females, are set up using some form of physical separation. This approach is used for mass spawning in fish and multisire joining in cattle. Animal behavior issues as well as genetic diversity and inbreeding issues are accommodated in such analyses.

### Management of progeny inbreeding

An individual cannot pass its merit for inbreeding to its progeny—inbreeding is not heritable (Kinghorn and Kinghorn 2021). Indeed, the current results show the potential danger in mate allocation to minimize progeny inbreeding without considering longer-term impacts. In a highly effective *IVF* program, selecting just 1 male plus 1 female as the only parents of the next generation because they are totally unrelated gives noninbred progeny—however the longer-term impact on inbreeding and diversity would be devastating. Conversely, when breeding for the highest possible progeny inbreeding in a species producing litters of progeny, a full-sib male mate will be chosen for each female. This means that many more males will be used than under normal circumstances, giving lower inbreeding in the long term, despite aiming high in the short term. The gray lines in Figs. 3 and 4 tell a third such story, and help to remind us not to be fooled into security by just allocating mates to minimize progeny inbreeding.

Results in this paper show a clear advantage to weighting against progeny $\bar{F}$ compared with the common practice of rejecting matings over an “*F* upper limit” (Traylor-Holzer 2011, p. 89). There are reasons for this:

1. Using *F*_*limit*_ gives precedence to a nonheritable transient component (progeny inbreeding) over a fully heritable accumulating component (mean coancestry or *MK*_*i*_). However, by using a relative weighting on $\bar{F}$, these 2 components compete across the full range of their values, giving better use of the available information and resources. Using *F*_*limit*_ probably eliminates some key half-sib matings that the *CAM* approach promotes.
2. *F*_*limit*_ operates as a hard constraint to exclude matings that might otherwise favor any other factor, not just coancestry. This alters the balance in opportunity and change in these other factors. Given the privilege of hard constraint, *F*_*limit*_ can compete harder against trait emphasis in the objective function, with a lower trait response but lower coancestry seen in 2023/2024 (Fig. 4, but not seen without trait emphasis in Fig. 3). Although inbreeding is not heritable, this lowered coancestry can happen because a rejected highly related pair above *F*_*limit*_ contributes more coancestry to the next generation than an accepted lowly related pair below *F*_*limit*_. However, this short-term coancestry improvement when using *F*_*limit*_ is at the expense of higher long-term coancestry due to reduced emphasis on *CAM*.
3. In our immigration scenario, *CAM* is invoked by allocating mates to maximize variance in progeny *MK*_*i*_ for the selected candidates. A mating pair that is high–high (or low–low) for *MK*_*i*_, could be highly related or lowly related. Applying a small weighting to $\bar{F}$ will favor the lowly related pairs, even if they are above *F*_*limit*_, and shift mate allocations in that direction as long as there is sufficiently little impact on overall variance in progeny *MK*_*i*_. This is what has given good outcomes for *CAM* treatments that put an appropriate weighting on $\bar{F}$.
4. A low–low *MK*_*i*_ mating pair with a low progeny $\bar{F}$ is probably of higher value than such an unrejected pair with a somewhat high $\bar{F}$, because they are likely to represent greater genetic diversity—their low *MK*_*i*_ values will be for somewhat different reasons. The *F*_*limit*_ approach will not exploit this benefit.
5. Valuable low–low *MK*_*i*_ pairs would tend to be highly related, especially in smaller populations, and yet be rejected for allocation because of a limit on progeny $\bar{F}$. This might help explain the poor performance of *OCS*_*td*_*CAM.F*_*limit*_ (see Fig. 4), which does not permit such matings. Moreover, this reduces the minimum achievable coancestry in the next generation, which in turn increases the 90-degree reference point of the next generation frontier (see Fig. 1), making the 45-degree target more balanced toward trait response. This can be seen in the increased trait and coancestry trends for *OCS*_*td*_*CAM.F*_*limit*_ from about 2026 in Fig. 4 and Supplementary Figs. S2 and S4. Another type of treatment comparison for this case would involve dictating change in coancestry at each generation and observing trait response, as done by Kinghorn and Kinghorn (2021), or vice versa, both easily done.
6. In summary, all matings above *F*_*limit*_ are *equally* rejected, and all matings below *F*_*limit*_ are *equally* accepted, with no exploitation of the variation in progeny *F* within each of these 2 groups.

### Altering policy over time

Treatments used in this paper were applied without change across the testing phase of simulations (2021 to 2040). This is probably not optimal. For example, using low weightings on progeny inbreeding in early years would help avoid the damaging effect of crossing immigrants into the established population, but after equilibrium of these genetic groups has been approached, a higher weighting against progeny inbreeding might be warranted, as might a lower weighting on *CAM*. Practitioners could test such variations in policy over time, perhaps simulating ahead from the base of real existing data. It is much simpler to treat each prevailing selection and mating cycle separately, and investigate possible outcomes on each occasion (Fig. 1). However, in this case, the practitioner needs to be well aware of what patterns of results promote desired outcomes for the longer term.

### Effects of inbreeding depression

As for Cowling et al. (2017), inbreeding effects on genetic variance were accommodated, but no effect of inbreeding depression was simulated when generating trait values in PopSim. If this were to be done it is likely to improve the superiority of treatments employing *CAM*, as these treatments resulted in considerably lower inbreeding coefficients.

### Use of genomic information

The current work has simulated pedigree relationships and used these to calculate coancestries and inbreeding coefficients. It would also have been possible to simulate genotypic data at many loci, and use that information for generating trait genetic values, and for estimating coancestries and inbreeding coefficients for making breeding decisions and/or evaluating outcomes. There is no major reason that this would impact conclusions, although calculating relationship matrices in a manner that allows *QTL* to have some influence could have some impact on outcomes (see Meuwissen et al. 2020). Many practical applications will have genomic information available to help manage diversity and inbreeding, which is especially useful when pedigree information is absent or dubious.

### Application to other scenarios

The key approach in this paper is the fostering of an immigrant population over some generations so that it can better serve the main population with its attribute(s). In the current case the attribute is genetic diversity. However, the same approach could be used for other attributes, such as notably high genetic merit for valuable traits, or gene edits to be introgressed from a small experimental population into a large breeding population.

### Comparison with other methods

Some breeding designs that aim to reduce inbreeding in the longer-term employ population division, with use of males cycling between subpopulations/families in some pattern (Caballero et al. 2016; Mucha and Komen 2016). The *OCS*_*x*_*CAM* method could be seen as a generalized and possibly optimal version of this approach, implemented at the level of individuals rather than at the level of groups. But there is a key difference: The cycling designs aim to avoid progeny inbreeding for a number of generations ahead, whereas *CAM* aims to preserve “high diversity” genetic material for use over a number of generations ahead.

Different implementations of the generalized method employed in this paper relate quite closely to methods that are widely used in conservation and zoo-animal breeding. Table 2 illustrates this.

**Table 2.** A comparison of methods for selection and mating.

Methods described by Ivy and Lacy (2012)	Nearest equivalent using the method underlying current paper
Static *MK* Selection = Pair at a time selection on low *MK* among all *candidates*.	*OCS* for low coancestry selecting 1 pair from the list of *candidates*, with repeat analyses as required.
Dynamic *MK* Selection = As above, but with *MK* recalculated after each pair selection, prospective progeny having been added to the pool for *MK* calculation.	As above, selecting among *candidates*, but with previous matings decisions included as Committed Matings^a^.
Ranked *MK* Selection = From a list of candidates, the highest *MK* individual is moved to a sex-specific list. *MK*s are recalculated among remaining candidates then the highest *MK* candidate of the opposite sex is moved to its sex-specific list. This is iterated, moving candidates to the tops of the sex-specific lists, but with accommodation of unequal sex ratio, until the list of candidates is empty. Mate allocations starting at the tops of the 2 sex-specific lists proceeds until the target number of matings is met.	The same, but using *OCS* for high coancestry selection into the sex-specific lists. As with Ranked *MK*, *MK*_*i*_ calculations start with all *candidates* and progress over mating decisions to end at the top *selected* individuals alone.
Simultaneous *MK* Selection = A portfolio approach where all selection and mating decisions are made simultaneously. Objective function includes *MK* and *F*.	Standard mate selection implementation resulting in *OCS* plus weighting on progeny inbreeding (viz. treatment *OCS*_*d*_*R.F*_0.01_), with coancestry relating to *selected* individuals only.

^a^Committed Matings are matings that have usually been made already to produce pre- and postnatal progeny that are too young to be selection candidates, and other unavailable animals. Committed Matings are forced to be part of the mating list solution in addition to new matings to be made (Kinghorn 2018).

The Static, Dynamic and Ranked *MK* Selection methods all pair the male and female with the lowest *MK*s, followed by the male and female with the next lowest *MK*s, etc. This preferentially pairs immigrant animals in the current scenario, and should produce results similar to those from *OCS* with *CAM*, but only for cases not involving management of progeny inbreeding. However, these *MK* Selection methods all involve sequential decision-making, and are therefore generally not optimal, as later decisions can override the validity of decisions fixed earlier on:

- Under Static *MK* Selection, candidates that are not part of the final solution are used in *MK* evaluations that affect decisions. This is important, as unselected *candidates* do not contribute to the population and should thus not be involved in *MK* calculation.
- Under Dynamic *MK* Selection, earlier pair selections are accommodated in later pair selection decisions, but candidates that are not part of the final solution still affect *MK* evaluations used for decisions.
- Under Ranked *MK* Selection, *MK* calculations for each mating pair relate to different subsets of the full population, where all *MK* calculations should relate to the final selected animals alone. For example, early-selected pairs do not accommodate later-selected candidates in their final *MK* evaluations. A simple example with an incorrect outcome is given in Supplementary Material—the best pair to mate is actually given the lowest priority under Static, Dynamic and Ranked *MK* Selection. However, in many cases that target diversity alone this suboptimality may have little impact.

The Static, Dynamic and Ranked *MK* methods are implemented in *PMx* software, and have probably performed well in practice, but it seems that the Simultaneous *MK* Selection method should give the best outcome among methods in column 1 of Table 2, as all *MK* values for solutions tested in the simulated annealing algorithm are based on all *selected* individuals, and their extent of use in the solution. Thus, unlike the other *MK* Selection methods, Simultaneous *MK* Selection is expected to get the correct result for the example in Supplementary Fig. S5, if it converges properly. Ivy and Lacy (2012) found that Simultaneous *MK* Selection was slightly outperformed by Ranked *MK* Selection, but that this was likely because the algorithm occasionally converged on a local rather than the global minimum of the evaluated function. As noted in Results, 170 pairs of analyses in the current paper converged to the same results. Management of convergence is described here: https://youtu.be/xs0qTmHHwNA.

Simultaneous *MK* implementation minimizes an objective function $\bar{MK}+\gamma \bar{F}$ (Ivy and Lacy 2012). Managing progeny inbreeding with a weighting rather than a fixed limit gives Simultaneous *MK* an added advantage over the other *MK* Selection methods. However, $\bar{MK}+\gamma \bar{F}$ is equivalent to treatment *OCS*_*d*_*R.F*_*γ*_ in this paper, and using *γ* = 1.0 or 0.1, this resulted in much higher coancestry and progeny inbreeding in the medium- and long term, compared with treatments using *CAM*, for reasons already noted. This could be rectified by adding *CAM* to the Simultaneous *MK* objective function.

Mating pair rejection at an “*F* upper limit” in *PMx* may have detracted from the benefit of *MK* Selection in some cases, as seen in this paper. On the other hand, an appropriate weighting on progeny $\bar{F}$ can add substantially to this benefit. There seems to be a case to consider providing such an option in the popular *PMx* zoo breeding software.

### Overlapping generations

The simulations modeled discrete generations, for simplicity of illustration. However, under overlapping generations, some “*non-selected animals are part of the future gene pool of the population, and they might very well be selected as breeders in a subsequent year. Similarly, animals that are too young to be considered as available breeders certainly can contribute to the future gene pool, but are generally not included in calculation of the optimal set of current breeders identified by OCS methods*.” (Anonymous referee, pers com).

This can be handled by giving current juveniles (including those in utero, plus other currently unavailable animals) essentially the same future contribution status as prospective new progeny from new matings, by forcing the “Committed Matings” that generated these current juveniles to be part of the solution. The analysis is expanded to cover not just the current candidates, but the full life-cycle of a complete generation surrounding these candidates, with the analysis solution forced to include all these Committed Matings in addition to the current matings to be decided. This gives more appropriate *MK*_*i*_ calculations for current candidates, as illustrated at https://youtu.be/JpcLgFaM0AA. This issue is most important for ongoing nonseasonal breeding systems where relatively few matings are made each week or so, with most adults not mated in any 1 wk. This gives very overlapped generations. Simulation of weekly matings over 4 yr in pigs gave a 28% lower increase in mean coancestry when using Committed Matings, compared with standard OCS on available candidates (Kinghorn 2018).

### Integration with other technical and logistical issues

The methods used in this paper have only been implemented at the full simultaneous/portfolio level, as in the bottom right entry in Table 2, but usually with many additional objective function components. A great advantage of this simultaneous mate selection approach is that it is highly configurable to accommodate many technical and logistical issues that impact decisions in breeding programs. Examples include:

- Control selections and mate allocations according to ­animal locations, quarantine/health risk on migration, animal age profiles, animal size compatibility issues, transport costs, etc.
- Favor some conservation/restitution of multiple wild subpopulation types, coat colors, etc., possibly following undesirable admixture, while managing overall diversity.
- Include selection for a Diversity EBV (Kinghorn and Kinghorn 2023) calibrated to the wild population, probably using genomic information. This makes the captive population better placed to improve genetic diversity in the wild population, or appropriate parts thereof, following reintroductions to the wild. This should only be undertaken where genetic diversity in the wild population is under threat, as it must outweigh any negative effects of undesirable genetic change under the captive environment.
- Reduce animal movements when assembling mating groups, as done in poultry.
- Dictate any desired mean change and/or corrective matings for other factors such as mating behavior, resistance to disease, reversion of unwanted selection while in captivity, and Mendelian genetic conditions. This paper has used a single trait as an example. Wherever possible, any trait selection should be targeted at performance in the wild, for example through use of genomic selection calibrated on phenotypes observed on wild animals in the natural environment.

With many such factors to manage it is valuable to explore the range of possible outcomes. This can be implemented by altering constraints and weightings during the analysis, in the light of results reported as the analysis proceeds. This can be seen in Fig. 1, by editing weightings and constraints, then “Update” the analysis on-the-fly.

It is also possible to make selection and mating decisions among immature juveniles, set up as separate groups within the analysis, not for immediate application, but to help accommodate possible future mating decisions when making current mating decisions, as well as to invoke early culling. These and many other issues are routinely managed using this simultaneous approach in the domestic animal industries.

## Supplementary material

Supplementary material is available at *Journal of Heredity* online.

Supplementary Fig. S1. Results for treatments targeting genetic diversity alone, and for simulation of initial inbred population to $\bar{F}=0.49$.

Supplementary Fig. S2. Results over years for treatments that place equal emphasis on genetic diversity and genetic gain, and for simulation of initial inbred population to $\bar{F}=0.49$.

Supplementary Fig. S3. Results for treatments targeting genetic diversity alone, and for simulation of initial inbred population to $\bar{F}=0.95$.

Supplementary Fig. S4. Results over years for treatments that place equal emphasis on genetic diversity and genetic gain, and for simulation of initial inbred population to $\bar{F}=0.95$.

Supplementary Fig. S5. Simple example whereby Ranked *MK* Selection leads to an incorrect outcome. See text for details.

Supplementary Fig. S6. Pedigree diagrams for treatments *OCS*_*d*_*R.F*_1.0_ (upper diagram) and *OCS*_*d*_*CAM.F*_0.001_ (lower diagram). See text for details.

Supplementary Table S1. Summary results across initial population mean inbreeding coefficients. *F*_*best*_ relates to the weighting on progeny inbreeding that gave the best result. For *OCS.R.F*_*best*_, *best* = 0 in all cases, for reasons described in the text.

esad027_suppl_Supplementary_Material

esad027_suppl_Supplementary_Data_S1

esad027_suppl_Supplementary_Data_S2

## Data Availability

The software package used to find optimal mate selection solutions (Fig. 1) and the population simulation program (Fig. 2) are available for research use through the authors via https://matesel.com/Website/researchrequest. Data generated in the simulations are available on request from the authors.
