# Supplementary material for: Management of diversity and inbreeding when importing new stock into an inbred population
Source: J Hered. 2023 Apr 29;114(5):492–503. doi: 10.1093/jhered/esad027 (PMC10445517; doi:10.1093/jhered/esad027)

Results for populations starting at higher inbreeding levels

This section shows results for populations that started from higher inbreeding levels ($\bar{F}$≈0.49 and 0.95). As might be expected, results are generally stronger for higher initial inbreeding levels.

Figures as low as 33% in the top six rows of Table S1 show that *OCS* without *CAM* can result in a threefold increase in loss of diversity compared to the wild population. The 59% result for initial $\bar{F}$=0.34 is more realistic, but still substantial. An aberrant figure in Table 1 is 132% for mean inbreeding in the top row. This is seen in figure 4 and explained in the main text. The impact of trait selection to give this result under $\bar{F}$=0.34 is damped at $\bar{F}$ = 0.49 and especially at 0.95, probably because of the more dominant effect of diversity issues, with high variance of coancestries in these populations. The bottom three rows in Table S1 show that placing a limit on progeny inbreeding resulted in mean coancestry and inbreeding between 26% and 102% higher than where an appropriate weighting against mean progeny inbreeding is used. However, the highest figures are influenced by an altered balance between diversity and trait response, as discussed in the main text, such that the 102% increase in inbreeding is associated with at 7% increase in response.

Figure S4 shows a slow start to selection response in the trait. With a mean inbreeding coefficient of 0.95 there was little genetic variation at the time of importing 10 new individuals, and it would take some generations of segregation for the new variation that they bring to be available for selection across many candidates. As a less important effect, given unknown pedigree for the immigrants, accuracy of *EBVs* would take a few generations to build up.

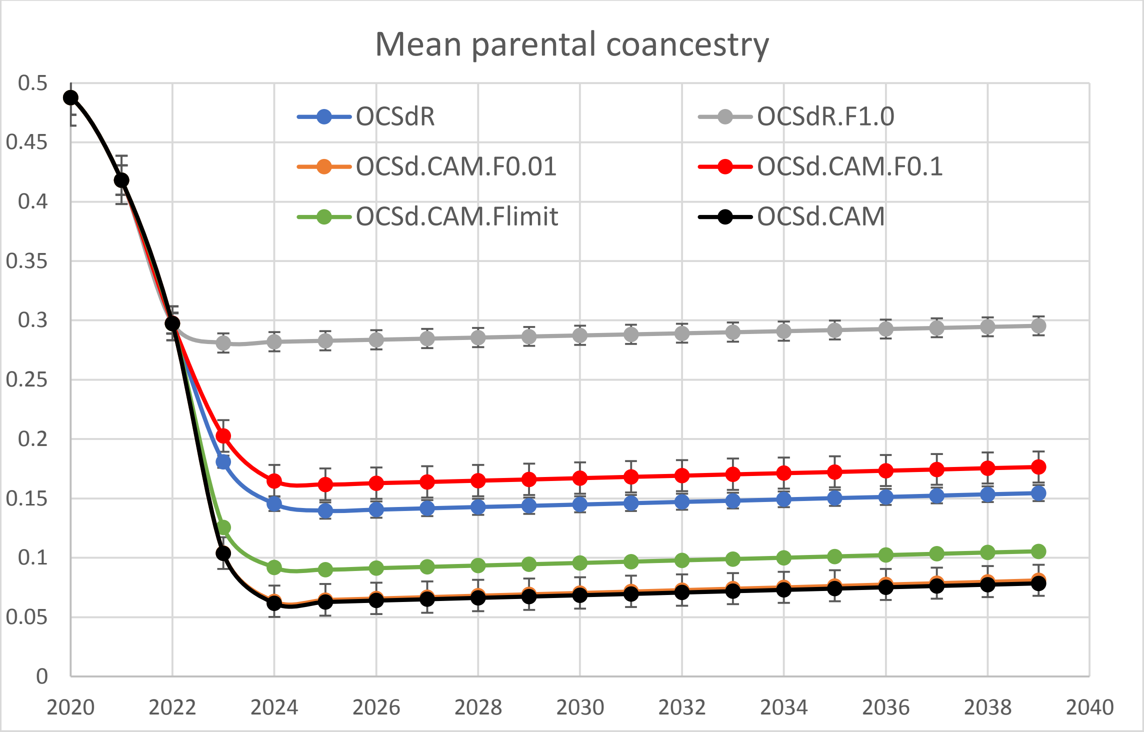

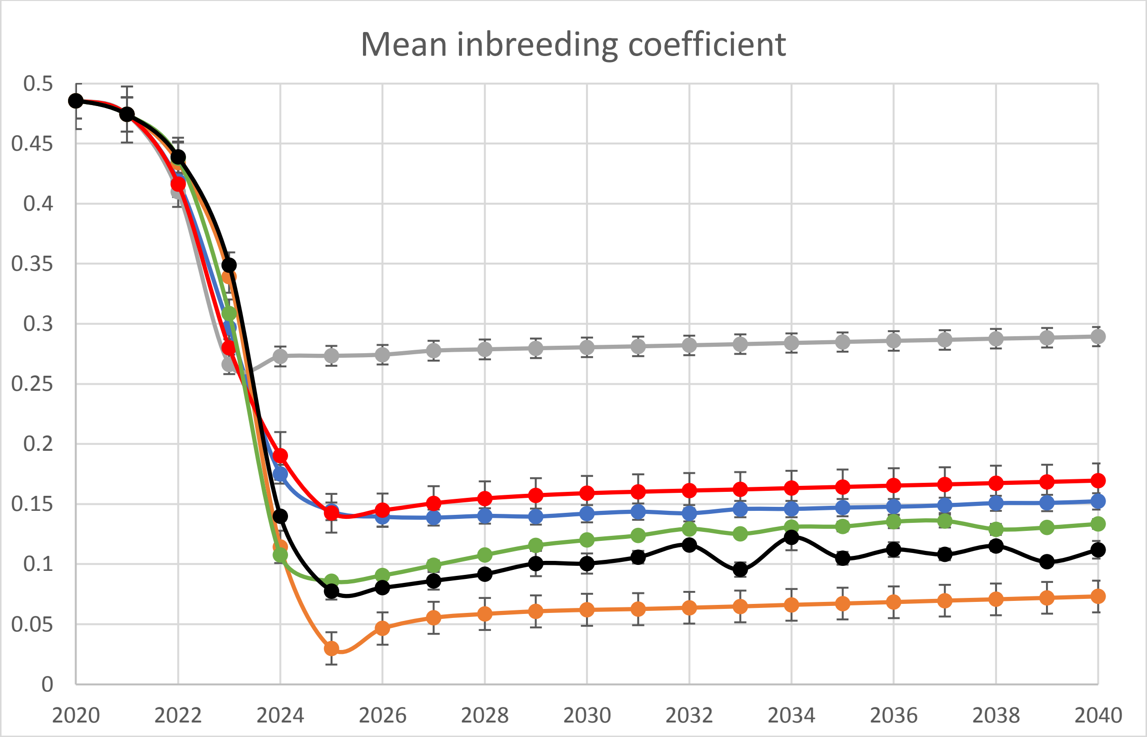

Figure S1. Results for treatments targeting genetic diversity alone, and for simulation of initial inbred population to $\bar{F}$=0.49.

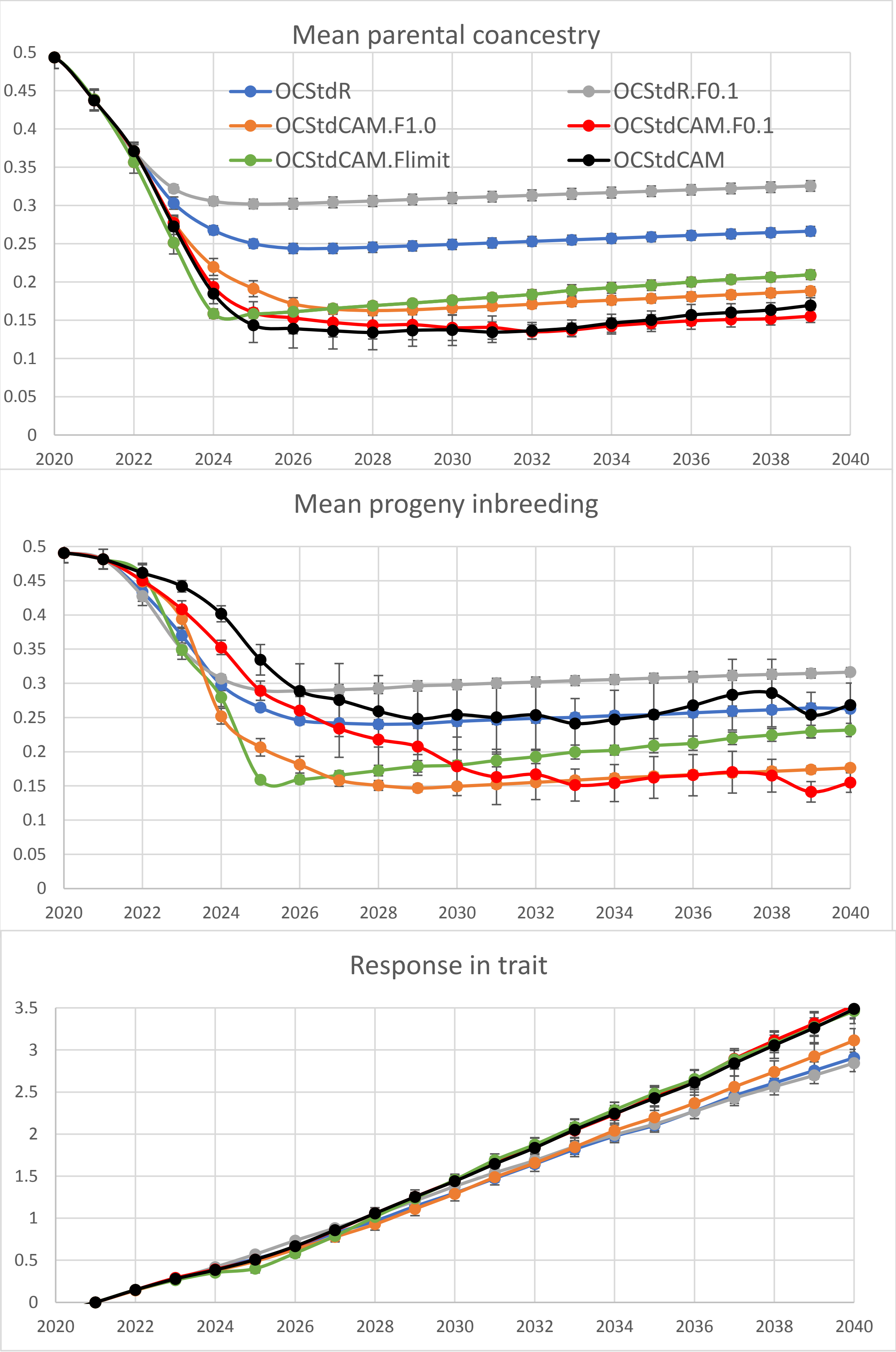

Figure S2. Results over years for treatments that place equal emphasis on genetic diversity and genetic gain, and for simulation of initial inbred population to $\bar{F}$=0.49.

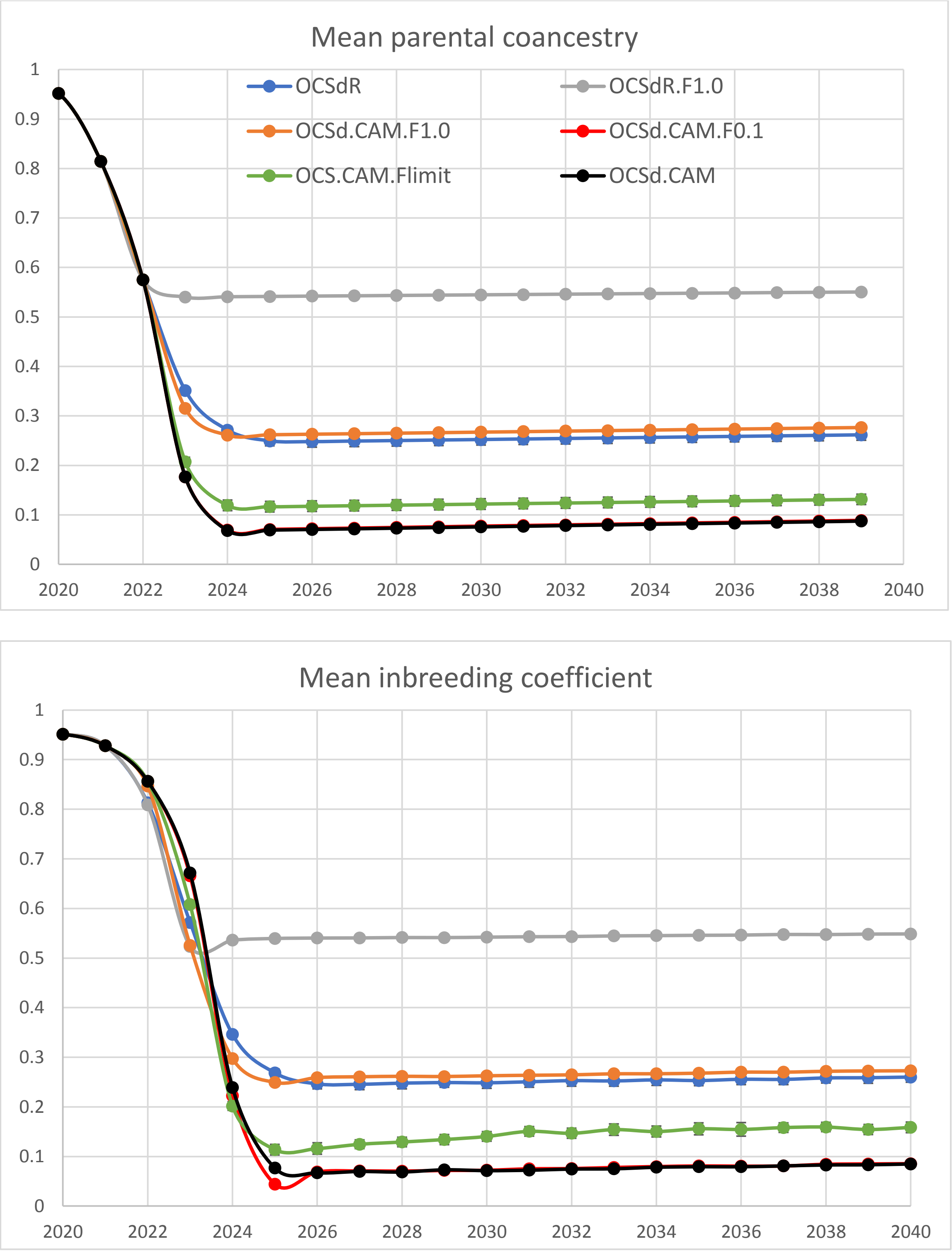

Figure S3. Results for treatments targeting genetic diversity alone, and for simulation of initial inbred population to $\bar{F}$=0.95.

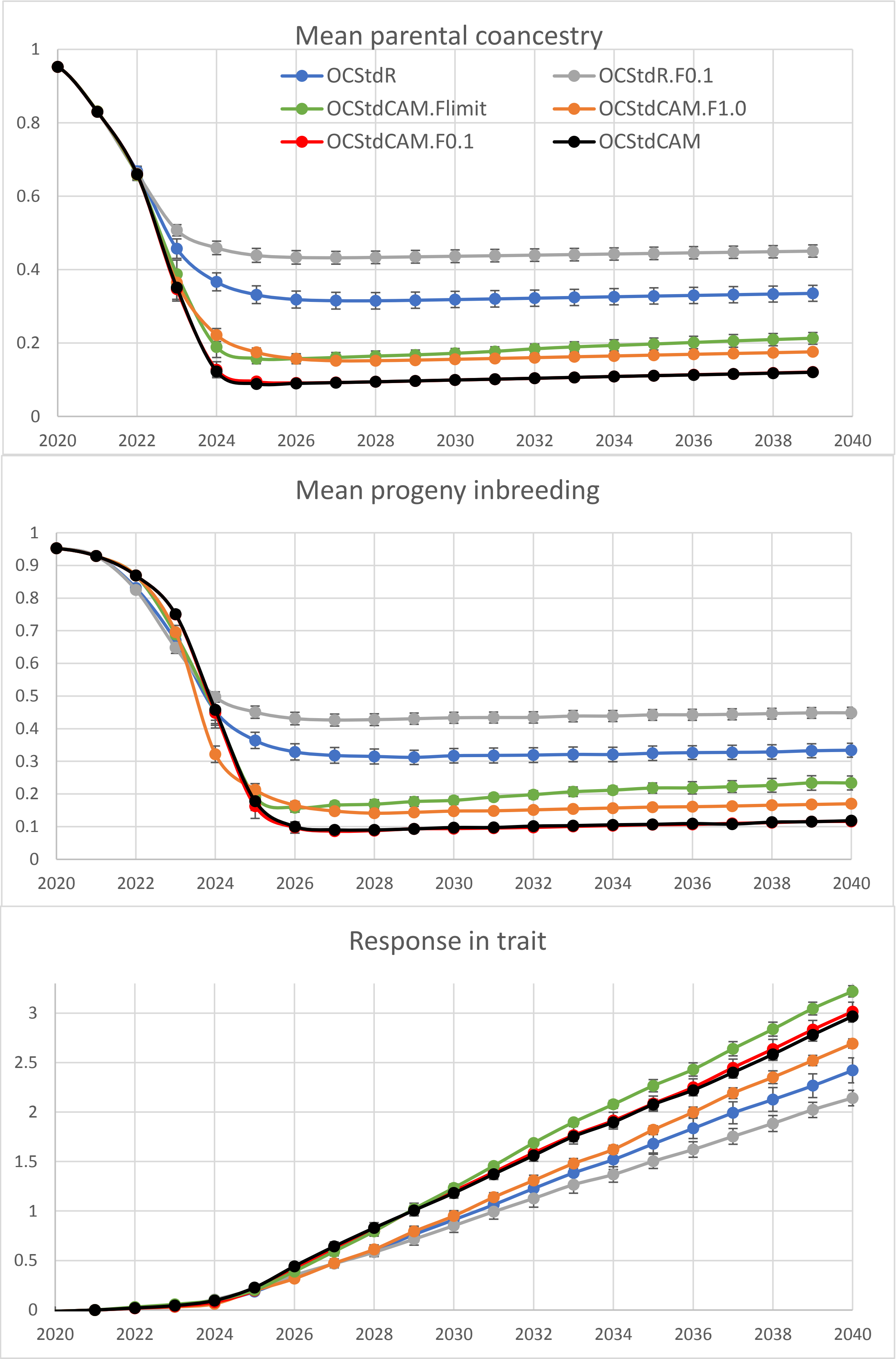

Figure S4. Results over years for treatments that place equal emphasis on genetic diversity and genetic gain, and for simulation of initial inbred population to $\bar{F}$=0.95.

Table S1. Summary results across initial population mean inbreeding coefficients. F_{best} relates to the weighting on progeny inbreeding that gave the best result. For *OCS.R.*F_{best} , *best*=0 in all cases, for reasons described in the text.

	Initial $\bar{F}$	Mean Coancestry		Mean Inbreeding		Response
		Diversity alone	Diversity +Trait	Diversity alone	Diversity +Trait	Diversity +Trait
OCS.CAM.F_0 as % of *OCS.R.*F_0	0.34	59	82	87	132	112
	0.49	51	64	73	102	120
	0.95	33	36	33	35	123
OCS.CAM.F_{best} as % of *OCS.R.*F_{best}	0.34	59	72	55	69	112
	0.49	51	58	48	59	121
	0.95	33	36	33	35	125
OCS.CAM.F_{limit} as % of *OCS.CAM.*F_{best}	0.34	126	142	172	168	99
	0.49	134	135	182	162	98
	0.95	151	177	187	202	107

Example where the Ranked MK Selection algorithm gives an incorrect result.

Consider that a single pair mating is to be made from all the animals in the pedigree shown in Figure S5. Males are odd-numbered and females are even-numbered. Relationships are dictated by the pedigree, with the exception that animals 3 to 10 inclusive are from a single population and are lowly related to each other, with kinships of value k < 2.27% between each pair. Animals 1 and 2 were unrelated immigrants in the previous generation. The optimal solution is to mate male 1 with female 2, as they are the only totally unrelated animals.

However, under Ranked MK selection, animals 1 and 2 are given the lowest priority (also under Static and Dynamic MK selection): they are the first two animals to be placed in the sex-specific lists described in Table 2, because they have some close relatives and they have the highest MK values (8.33%, compared to 4.79% for animals 3 to 10, and 7.94% for animals

11 to 18, at k = 1%. See *FigS5Results.xlsx* for the worked example). The method used in this paper selects animals 1 and 2 as the single mating pair.

Figure S5 has 4 matings per immigrant whereupon $MK = \bar{F}$ in the main population has to be below 1/44 = 2.27% for animals 1 and 2 to be the first rejected. As shown in *FigS5Results.xlsx*, this figure increases asymptotically to 1/12 = 8.33% as the number of matings per immigrant increases. This is well below $\bar{F}$ = 34% generated in the simulations in this paper.

Where more than one mating pair is to be selected, animals 1 and 2 remain the lowest priority for selection under Ranked MK Selection. The optimal solution, found with the method used in this paper, and checked in *FigS5Results.xlsx* by exhaustive searching, includes animals 1 and 2 in all solutions except where 8 matings are required. For 1 to 7 matings required, Ranked MK Selection gives up to 20.6% higher mean kinship for these targets, as seen in sheet "Ranked MK Selection results" of *FigS5Results.xlsx*.

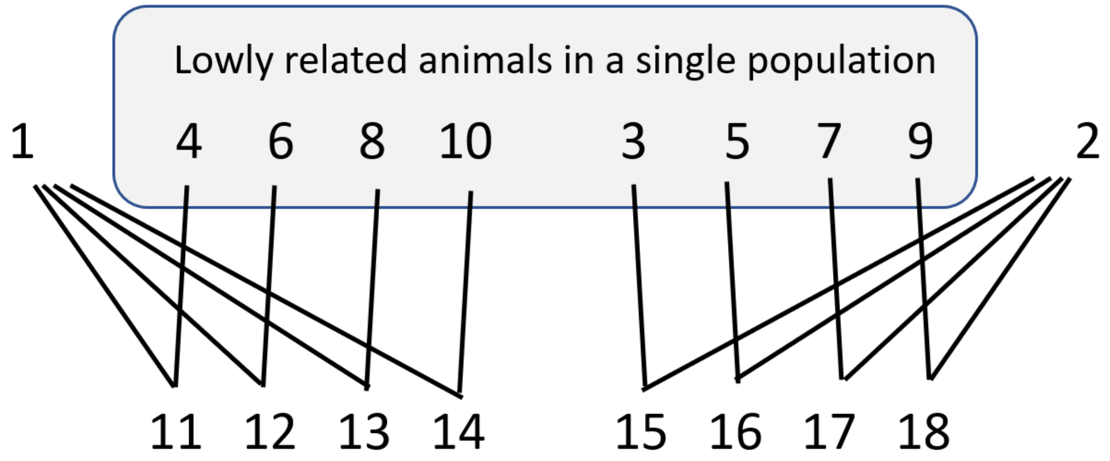

Figure S5. Simple example whereby Ranked MK Selection leads to an incorrect outcome. See text for details.

Scaling of objective function components

The impact of weightings applied under the treatments described depends on scaling of the objective function components involved, and this is described here for researchers using a different software implementation. The scale and range of coancestries and trait EBVs can have a major impact on what weightings should be used to target a desired outcome across all issues in the objective function. For the current method, parental coancestry and progeny index (an index of predicted progeny EBVs across traits, or genetic response in the single trait in our example) are treated in unison, with a single scale of value ranging from 0 to 1 representing both these components, and an implied weighting of 1 at all times. For the current constraint (solutions cannot be less than target degrees (***TD***) of 45 degrees) this value is:

$$\frac{G - G_{min}}{Cos(TD) * (G_{max} - G_{min})}$$

… where G is the progeny index of the solution, and G_{min} and G_{max} are the minimum and maximum possible progeny index values, at 90 and 0 degrees respectively. If the frontier curve were perfectly circular, this value would range from 0 at G_{min} to 1 at the maximum mean progeny index at the prevailing *TD*. In practice, the maximum possible value deviates from 1 by a small amount, as the shape of the frontier curve depends on the relationships, index values and usage constraints for the prevailing candidates. Solutions below *TD* = 45 degrees are illegal and rejected by the algorithm. This pattern applies wherever *TD* <= 45. For *TD* > 45, the function value switches to favour low parental coancestry, as follows:

$$\frac{C_{max} - C}{Sin(TD) * (C_{max} - C_{min})}$$

… where C is parental coancestry, with constraint to exclude solutions *above TD*. In this case, function value ranges 0 at C_{max} to 1 at the minimum parental coancestry at the prevailing *TD*. This means that for the 90-degree treatments used in this paper, all solutions are <= 90 degrees and so progeny index does not play a role, and low parental coancestry is favoured without constraint.

Progeny inbreeding and progeny coancestry were not scaled, as these are conveniently on a scale of 0 to 1. This means that for treatments involving F_y (weighting y on mean progeny inbreeding) the result for mean progeny inbreeding is simply multiplied by y to give that component of the overall objective function.

Pedigree diagrams that help to illustrate method

Figure S6 shows partial pedigree diagrams for the first replicate simulations of treatments $OCS_dR.F_{1.0}$ (upper diagram) and $OCS_dCAM.F_{0.001}$ (lower diagram). These were generated using Pedigree Viewer (https://bkinghor.une.edu.au/pedigree.htm) with the TierInfo option applied to Year of Birth, in order to force animals into one tier in the diagram for each year. If this were not done, then immigrant animals would end up in the top tier, as founders. Red lines emanate from males and yellow lines from females. Both diagrams show just seven tiers, for the animals born in the seven years 2021 (top tier) to 2027 (bottom tier).

The single data field shown is the inbreeding coefficient (*F*) for each animal. This information is hopelessly overlapped and unreadable. However, these values are shaded by their magnitude (black at *F* = 0 to white at F = ~0.35), and sorted left-to-right. Zooming into these diagrams may help.

Animals at the left of Tier 2 from the top (2022) are the immigrant animals. As can be seen, they have no known parents. They have *F*=0 (black) and so do all their progeny, as expected.

Without *CAM* (above), matings are made to minimise progeny *F*, giving more *F*=0 progeny in 2023 compared to the *CAM* policy below. It can be seen that many immigrant females (yellow lines coming from the left of 2022 to the left of 2023) have been mated to native males (red lines coming from the right of 2022 to the left of 2023).

Under *CAM* (below), no red lines come from the right of 2022 to the left of 2023, as all immigrant females are mated to immigrant males. This is because they are too valuable to be 'diluted' with native male matings. However, each male can mate two females, and it can

be seen that excess male contributions are used to mate native females (yellow lines coming from the right of 2022 down to the left of 2023), helping with both diversity and Progeny F.

Under treatment *$OCS_dR.F_{1.0}$*, mean F is reduced markedly by 2024 ($\bar{F}$ = 0.199 vs 0.254 under *CAM*). However, CAM has invested in more highly concentrated immigrant material by that time, easily seen by the much greater variation in Progeny *F* (variation in the grey scale), compared to the upper diagram. By 2025 mean *F* under *CAM* is 0.085 versus 0.205 without *CAM*. The big difference between treatments in the level of grey in the last two years clearly shows the advantage of applying *CAM*.

Figure S6. (Next page) Pedigree diagrams for treatments *$OCS_dR.F_{1.0}$* (upper diagram) and *$OCS_dCAM.F_{0.001}$* (lower diagram). See text for details.

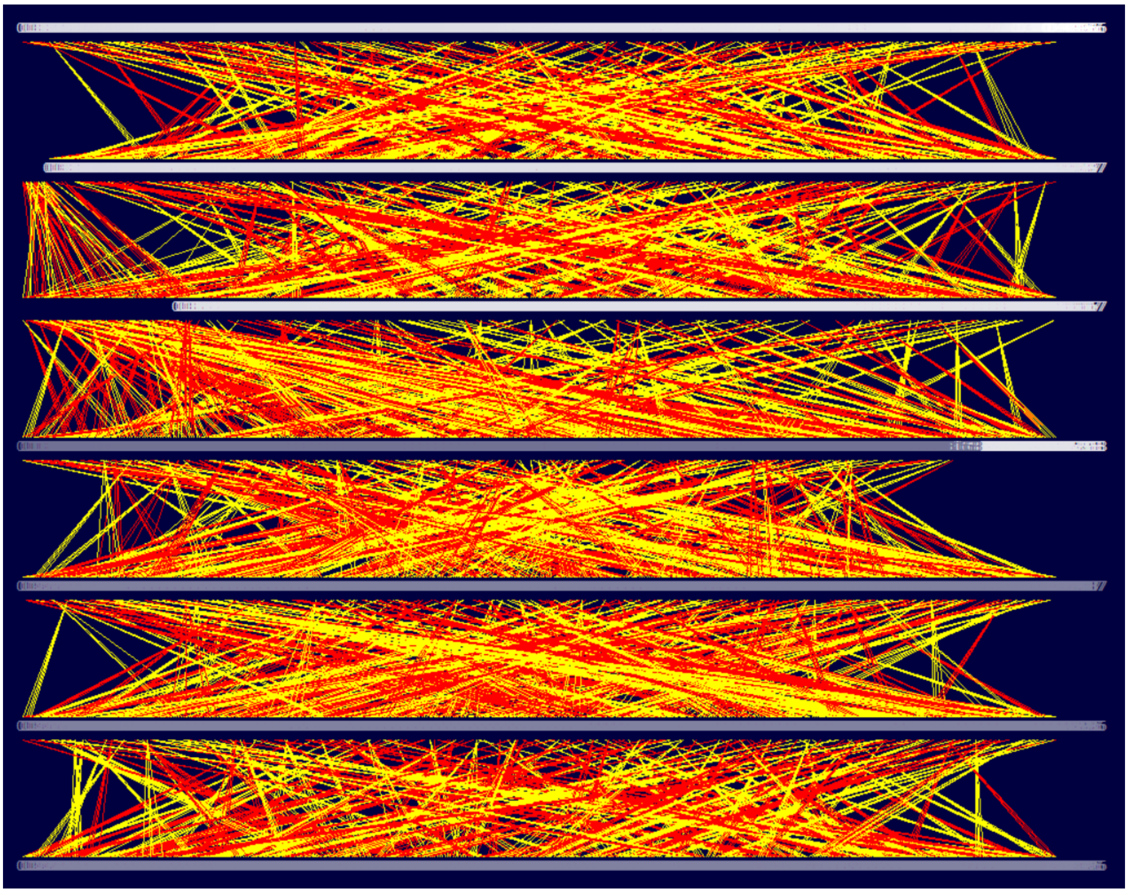

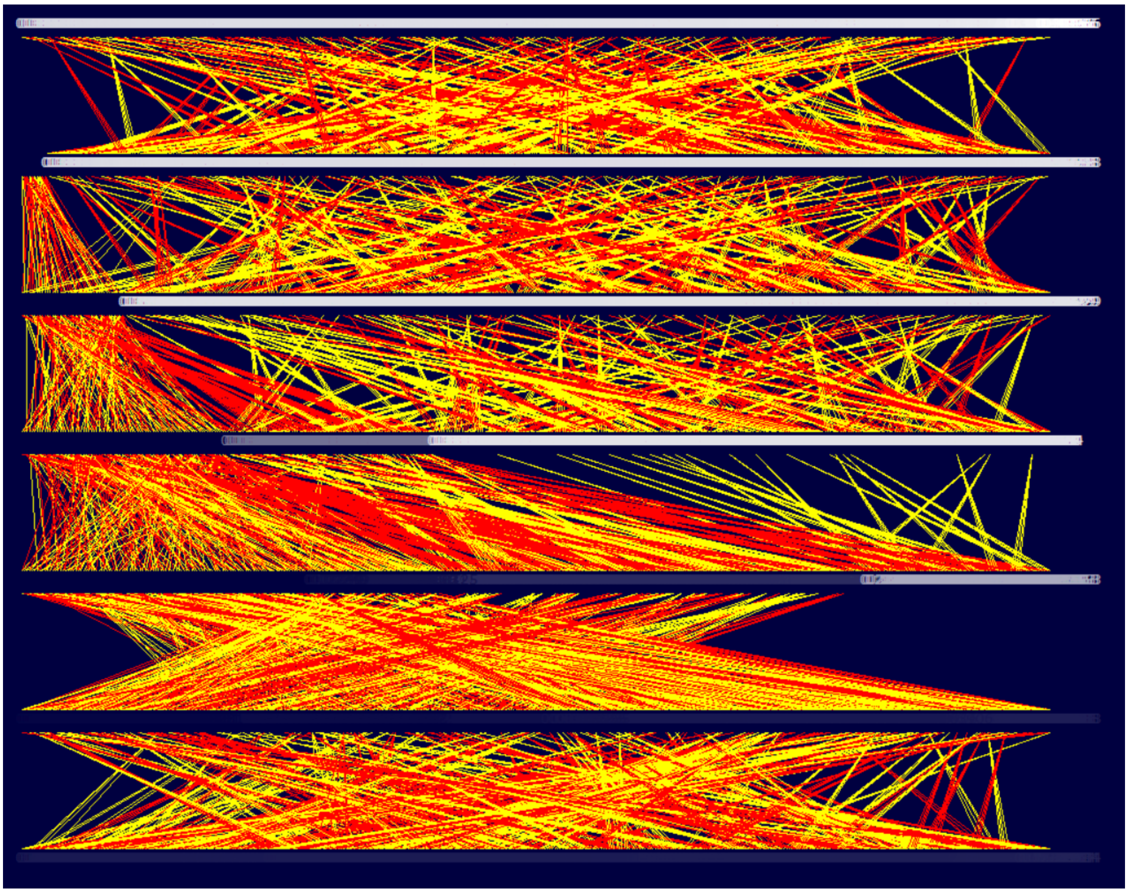

Supplement: esad027_suppl_Supplementary_Material [file esad027_suppl_supplementary_material.pdf]
